# Perforation of Meckel's diverticulum by an unusual foreign body: A case report and a review of literature

**DOI:** 10.1002/ccr3.9183

**Published:** 2024-07-29

**Authors:** Sara Elizabeth Milla Salguero, Enrique Adalberto Medina, Alejandra Hause Murillo, Eduardo Smelin Perdomo Domínguez

**Affiliations:** ^1^ Faculty of Medicine Universidad Católica de Honduras San Pedro Sula Honduras; ^2^ Department of Pediatrics, Hospital Mario Catarino Rivas Universidad Nacional Autónoma de Honduras (UNAH) San Pedro Sula Honduras; ^3^ Department of Pediatric Surgery Hospital Mario Catarino Rivas San Pedro Sula Honduras; ^4^ GIMUNICAH, Faculty of Medicine Universidad Católica de Honduras San Pedro Sula Honduras

**Keywords:** foreign body, Meckel's diverticulum, perforation, wood splinter

## Abstract

**Key Clinical Message:**

Perforation of Meckel's diverticulum (MD) is rare, particularly by foreign body. High index of suspicion and thorough intraoperative assessment is needed in patients undergoing surgery for acute appendicitis, specifically when appendix appears normal.

**Abstract:**

Meckel's diverticulum is the most common congenital anomaly of the gastrointestinal tract. While often asymptomatic, it can present with several complications. Perforation due to foreign body ingestion is rare but can have severe consequences if late diagnosis occurs. A 13‐year‐old male, initially suspected of acute appendicitis, was eventually diagnosed with perforation of MD by a wood splinter‐like foreign body after intraoperative assessment. Histological analysis revealed ectopic colonic tissue within the MD, a finding whose implications are not well understood, in contrast with the well‐established complications associated with ectopic gastric and pancreatic tissues. This case highlights the diagnostic challenges of MD, which can mimic acute appendicitis, emphasizing the need for high suspicion when faced with atypical clinical presentation such as foreign body‐induced perforation. Although surgical resection of asymptomatic MD remains controversial, we recommend a case‐specific approach based on risk factors to guide decision‐making on surgical resection for asymptomatic MD.

## 1 INTRODUCTION

Meckel's diverticulum (MD) is the most common congenital anomaly of the gastrointestinal tract that occurs as a result of the incomplete obliteration of the omphalomesenteric duct, leading to the formation of a true diverticulum of the small intestine.^1^ It is considered a true diverticulum since it involves all layers of the small intestine, arising from the antimesenteric border of the mid to distal ileum.^2^ Its prevalence ranges between 2% and 4% of the general population.^1^ MD diagnosis is often challenging since the condition usually remains asymptomatic and can mimic various diseases. The most common complications include intestinal obstruction due to intussusception or adhesions, ulceration, diverticulitis, and occasional perforation.^3^ Perforation represents a rare complication of MD. In more unusual cases, perforation can be due to the ingestion of a foreign body, which could have a poor prognosis if delayed diagnosis occurs. We present the case of a 13‐year‐old male patient with perforation of MD by a wood splinter, after initial presumptive diagnosis of acute appendicitis.

## 2 CASE REPORT

### 2.1 Case history and examination

A 13‐year‐old male patient presented to the emergency department with a 24‐h history of abdominal pain. He had no significant past medical history, medication use or previous surgeries. The pain was localized in the epigastrium, had a colicky nature, moderate intensity, and radiated to the right iliac fossa. It was not relieved at rest and was exacerbated by walking. Additionally, he had one episode of light brown, loose, and watery diarrhea. The patient denied having a fever, nausea, or vomiting. Upon admission, the patient exhibited normal vital signs. Abdominal examination revealed rebound tenderness localized to the right lower quadrant. Despite this finding, bowel sounds were present, and there was no evidence of guarding. Laboratory data showed hemoglobin at 14.9 g/dL, hematocrit at 44.1%, a white blood cell count of 24.2 × 10^3^/mm^3^, neutrophils at 86.6%, platelets at 338 × 10^3^/mm^3^, and a urinalysis without pathological findings. Based on these findings, an initial diagnosis of acute appendicitis was established.

### 2.2 Treatment, outcome, and follow‐up

**FIGURE 1:**
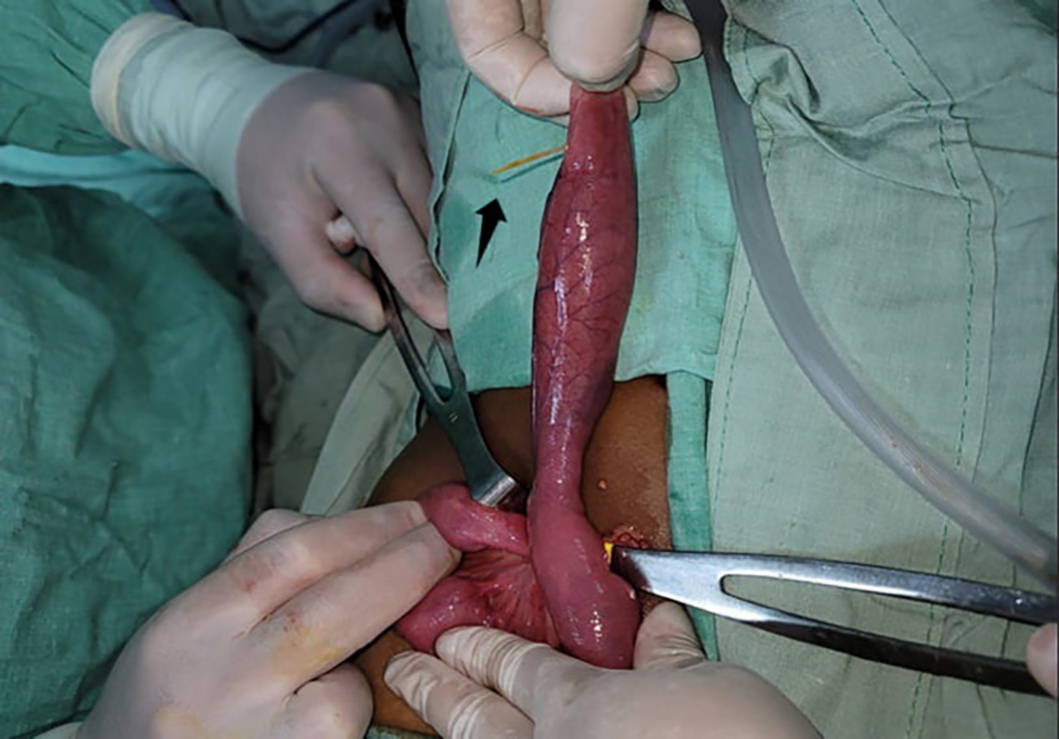
Intraoperative view showing Meckel's diverticulum perforated by a wood splinter (black arrow).

During the exploratory laparotomy, a cecal appendix with normal characteristics was observed, along with a moderate amount of purulent fluid in the pelvic cavity, leading to a prophylactic appendectomy. Subsequently, based on these findings, the distal ileum was assessed. A wide‐based MD was identified, located 80 cm from the ileocecal valve, perforated by a foreign body suggestive of a wood splinter (Figure 1). An intestinal resection followed by an end‐to‐end anastomosis was performed.

**FIGURE 2:**
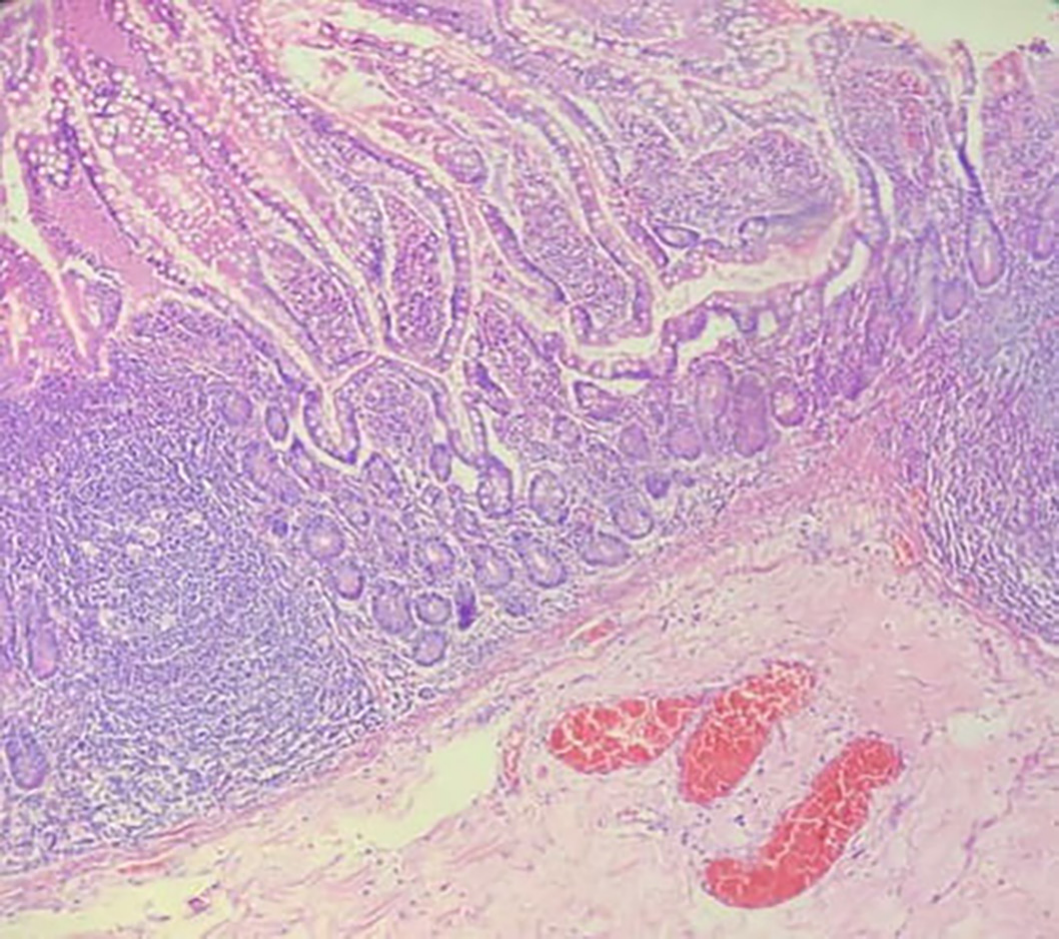
Histological analysis demonstrating ectopic colonic tissue, with surrounding inflammation and extravasated red blood cells.

Histological analysis of the surgical specimen revealed a small intestine segment measuring 5 × 2.5 cm with a 4 cm saccular lesion located 2 cm proximal to the closest surgical margin. This microscopic examination identified ectopic colonic mucosa (Figure 2) with goblet cells within the MD. There were significant areas of hemorrhage in the submucosa and serosa, accompanied by an acute and chronic inflammatory response, with lymphocytes being predominant.

After surgery, a clear liquid diet was started on the second postoperative day, and a soft diet on the fourth postoperative day, with a gradual return to normal diet over 5 days. The clinical recovery course was uneventful. The patient was discharged after 10 days of hospitalization without postoperative complications. One‐month follow‐up revealed the patient was essentially well and remained asymptomatic. No further radiological images were deemed necessary.

## 3 DISCUSSION

We described a patient with perforation of MD by an unusual foreign body, a wood splinter, in a 13‐year‐old male patient, initially mimicking symptoms of acute appendicitis, associated with the presence of ectopic colonic tissue.

Meckel's diverticulum is the most common congenital malformation of the gastrointestinal tract. It arises from the persistence of the omphalomesenteric duct beyond fetal development. The prevalence of MD is approximately 2%; however, Zani et al. found a prevalence of 1.2%.^4^ The overall lifetime risk of complication is generally recognized to be 4%, with a male‐to‐female ratio between 1:1.8 and 3:1.^5^ However, some studies have reported the incidence of complications rising to as high as 16%.^1^ In adults, Yamaguchi et al., described the incidence rate of complications in a retrospective study of 600 patients, as follows: intestinal obstruction 36.5%, intussusception 13.7%, inflammation 12.7%, hemorrhage 11.8%, perforation 7.3%, and foreign body 0.5%.^6^ Chen et al., in a retrospective analysis of patients, aged from 1 day to 15 years, observed that 35% presented with rectal bleeding or melena, 20% with Meckel's diverticulitis or perforation, 14% with intestinal obstruction, and 12% with intussusception.^7^ While the studies by Yamaguchi et al. and Chen et al. provide insight into the typical presentation of complications in adults and pediatric patients respectively, it is essential to understand that these complications are not exclusively age‐specific. Some pediatric patients may predominantly present with intestinal obstruction as their primary clinical feature, while some adults might primarily manifest with gastrointestinal bleeding.^8^, ^9^ Other rare complications include tumors, inversion of MD, torsion, volvulus of ileum around MD, fibrous cord, Littre's hernia, and vesico‐diverticular fistulae.^1^, ^10^

Various imaging studies have been described for the diagnosis of MD, each imaging modality with specific indications and limitations. Ultrasound is emerging as an alternative, particularly for gastrointestinal bleeding, though its sensitivity can be limited by operator‐dependence, gas accumulation and thick abdominal wall. Recent studies have showed high sensitivity and specificity in diagnosis of bleeding MD in pediatric patients.^11^, ^12^ Scintigraphy, using Technetium‐99m (Tc‐99m) pertechnetate, is preferred in pediatric patients with lower gastrointestinal bleeding due to its high sensitivity and specificity of 0.80 (95% CI 0.73–0.86) and 0.95 (95% CI 0.86–0.98) for detecting ectopic gastric tissue within MD in children.^13^ In adults, however, its diagnostic accuracy is lower (20%–60%).^14^ Advanced endoscopic modalities, like capsule endoscopy and device‐assisted enteroscopy, offer valuable insights, particularly in cases of suspected small bowel bleeding.^15^, ^16^ Mesenteric angiography is a potentially significant diagnostic tool, particularly when other imaging studies fail to diagnose MD, aiding in the diagnosis through visualization of the vitelline artery.^17^, ^18^ Computed tomography scans can identify MD as a fluid or air‐filled pouch, especially useful when appendix pathology is excluded, but have low sensitivity for asymptomatic MD.^19^ Magnetic resonance enterography is also a viable option, accurately determining MD location and complications without radiation exposure.^20^ Overall, the choice of imaging depends on clinical presentation, with a need for further studies to establish the definitive role of each modality in MD diagnosis.

The variability in clinical presentation underscores the importance of a comprehensive differential diagnosis irrespective of age. The clinical presentation of MD can often mimic that of acute appendicitis, particularly when complications arise. Our case was highly suggestive of acute appendicitis, considering the localized tenderness in the right lower quadrant, the patient's age, and laboratory findings. Similarly, Anyfantakis et al. reported a case involving a 4‐year‐old male who exhibited the same clinical manifestations with an initial presumptive diagnosis of acute appendicitis, however, upon laparotomy, a MD perforated by a wood splinter was observed, while the appendix appeared normal.^21^

Perforation in MD can lead to several serious complications; therefore, early identification and management are crucial. Patients often present with generalized peritonitis, which can subsequently progress to bacteremia and escalate into sepsis, potentially resulting in multiorgan dysfunction. Additionally, patients may develop local intra‐abdominal abscesses or phlegmon formation.^22^ Moreover, fistula formation represents a significant complication, creating abnormal connections between the diverticulum and other organs.^23^ These complications elevate morbidity and mortality rates, necessitating intensive monitoring and prompt intervention to prevent fatal outcomes.

Various foreign bodies, chicken bones, melon seeds, peanuts, bay leaf, toothpick, batteries, needles, pins, wood splinters, and especially fish bones—which account for 55% of cases—have been implicated as causes of MD perforation in both children and adults.^24^, ^25^ Notably, patients often do not recall the ingestion of these foreign bodies. The majority of ingested foreign bodies will pass spontaneously, as pre‐endoscopic series have shown that 80% or more would naturally pass through the gastrointestinal tract without any medical intervention.^26^ MD perforation by wood splinters are extremely rare. It is worth noting that Rosswick documented six cases of MD perforated by wood splinters within an analysis of 50 cases, however, the specific clinical presentations were not detailed.^27^

Ectopic tissues are found in approximately 55% of MD, with gastric tissue being the most prevalent, observed in 60%–65% of cases, and pancreatic tissue identified in about 5% of cases.^28^ Although gastric and pancreatic tissues are commonly implicated, other tissues such as duodenal, colonic, Brunner's glands, hepatobiliary tissue, and endometrial mucosa have also been identified.^28^ The underlying mechanism by which heterotrophic mucosa occurs within MD is related with the pluripotent nature of the cells that line the omphalomesenteric duct, capable of differentiating into any type of tissue.^29^ However, this hypothesis doesn't explain the predominance of one type of tissue over another, specifically gastric and pancreatic tissue. It has been proposed that the formation of ectopic pancreatic tissue is triggered by misplaced tissue from migration and fusion of pancreatic buds or potentially due to disrupted molecular signaling, specifically the loss of sonic hedgehog gene.^30^, ^31^ Regarding gastric heterotopia, aberrant differentiation of pluripotent endodermal stem cells, might lead to the formation of gastric ectopic tissue.^32^ Burjonrappa et al. proposed an interesting hypothesis, suggesting that the differentiation of pluripotent cells into specific types of tissue, might be influenced by nutritional and growth factors, guiding the phenotypic expression of these cells in the early post‐natal development.^29^

The potential risk of malignant neoplasms in ectopic tissue is considered rare, with an incidence rate of about 0.5%–5.1%.^33^, ^34^ Among the predominant types of malignant neoplasms, neuroendocrine tumors are the most common (63.2%), followed by gastrointestinal stromal tumor (GIST) (10.5%), adenocarcinoma (5.3%), and pancreatic epithelial neoplasia (5.3%).^33^ Other less common malignant neoplasms have been identified, these include: intraductal papillary mucinous neoplasms, lymphomas, melanomas, and leiomyosarcoma.^34^ The development of neoplastic changes in MD might be influenced by various factors. The omphalomesenteric duct's pluripotent cells may create propitious conditions for malignancy, and the presence of heterotrophic tissue could represent a sign of improper molecular signaling, thereby increasing the risk for neoplastic development.^35^ Furthermore, gain‐of‐function mutations in the KIT gene have been associated with the occurrence of GIST in MD.^35^

Given the presence of ectopic colonic mucosa in our case, it may be feasible to considered that there is a minimal risk of developing tumors, such as adenocarcinoma of MD or goblet cell adenocarcinomas. While adenocarcinoma of MD can arise from ectopic colonic tissue, goblet cell adenocarcinomas, characterized by both mucinous and neuroendocrine differentiation, are extremely rare and usually occur in the appendix but can also be found in extra‐appendiceal locations like the colon.^34^, ^36^ The coexistence of these histological features in MD may potentially increase the risk of complex and rare malignant neoplasm originating from MD. We believe that further research will be needed to elucidate the link between ectopic colonic mucosa and complications arising from MD.

It has been well established that a symptomatic MD is typically managed through surgical resection; however, the surgical approach to incidentally detected MD is still controversial. Cullen et al., in their population‐based study, recommended surgical resection for incidentally detected MD regardless of age, reporting 2% short‐ and long‐term complications for asymptomatic MD, while complicated MD resection exhibited higher short‐ (12%) and long‐term (7%) complication rates.^37^ Moreover, MD‐related complications were greater in men than in women and a 6.4% overall lifetime risk of developing complications from MD was described.^37^ Several authors supported this recommendation, emphasizing that surgical resection for asymptomatic MD is safe due to a low morbidity and mortality rate compared to symptomatic MD.^7^, ^38^, ^39^, ^40^ In a retrospective study by Gezer et al., 40% of symptomatic MD exhibited life‐threatening complications, with post‐operative complications seen in 39% of patients with complicated MD.^41^ Contrary to previous suggestions that the macroscopic appearance of MD could predict heterotopic mucosa, specially thickening, the authors emphasized that incidentally detected MD should be removed regardless of its appearance. Furthermore, the presence of significant ectopic tissue could lead to a mass effect or feature a narrow neck, predisposing individuals to the development of intestinal obstruction or diverticulitis, thereby leaning towards surgical resection for asymptomatic MD.^42^ MD associated malignancy has emerged as a possible surgical indication for incidentally detected MD. Thirunavukarasu et al. identified MD as a high‐risk area for cancer in the ileum, with a risk of cancer 70 times higher for MD than other ileal locations.^43^ This supports the recommendation for surgical resection of asymptomatic MD, as the benefits of resection outweigh the risks of leaving it in situ. Bona et al. reported a case of a carcinoid tumor within a MD incidentally detected and removed during laparoscopic inguinal hernia repair. They emphasized that simple laparoscopic tangential resection with an endostapler is a viable and safe procedure, particularly in healthy individuals.^44^ From an oncological point of view, and considering the low risk for morbidity and mortality, several authors have advocated for surgical resection of incidentally detected MD.^45^, ^46^, ^47^

While the aforementioned studies strongly support surgical resection of asymptomatic MD, several authors have argued against resection of incidentally detected MD. Soltero et al. estimated the lifetime risk of complications to be 4.2%, with risk decreasing with age. They also concluded that 800 surgical resections would be necessary to prevent one death.^48^ This estimation aligns with Zani et al.'s findings, which reported that 758 surgical MD resections would be required to prevent a single death from MD.^4^ Additionally, resection of asymptomatic MD is associated with a significantly higher complication rate (5.3%) compared to leaving it in situ (1.3%), and patients undergoing surgical resection exhibit an increased risk of developing intestinal obstruction.^4^ In contrast, no complications were reported in long‐term follow‐up studies on patients with MD left in situ.^4^ Moreover, Peoples et al. argue against surgical resection of asymptomatic MD, as it is associated with a higher morbidity (4.1%) and mortality (0.2%) rate compared to symptomatic MD resection, with rates of 0.2% and 0.04%, respectively.^49^ Stone et al. argued against the incidental removal of asymptomatic MD, especially in women, as their study revealed that female patients were significantly less likely than male patients to exhibit symptoms, suggesting that routine resection may not be justified in this population.^50^

Other authors have advocated that decision‐making should be considered based on high‐risk individuals. Park et al. proposed four criteria: (1) patient age younger than 50 years; (2) male sex; (3) diverticulum length greater than 2 cm; and (4) ectopic or abnormal features within a diverticulum, all associated with symptomatic MD.^51^ The study found that meeting one criterion resulted in a 17% overall proportion of symptomatic MD, while meeting two, three, or all four criteria corresponded to proportions of 25%, 42%, and 70%, respectively. They suggested that surgical resection of asymptomatic MD could be considered if they fulfill any of these four criteria. McKay also identified patients under 50 years to have an increased risk of symptomatic MD and heterotopic mucosa, thus leaning towards surgical resection of asymptomatic MD in these patients.^52^ Robijn et al. proposed a risk score system to assess whether asymptomatic MD should undergo resection, based on criteria such as male sex, patients younger than 45 years, MD size greater than 2 cm, and the presence of a fibrous band.^53^ A score of 6 or more points, suggested surgical resection. Other authors recommended surgical resection of either symptomatic or asymptomatic MD in patients younger than 8 years, as there is an increased risk of morbidity in this group.^54^

Correlating with our case, MD perforation is unusual and only 1.6% of total gastrointestinal perforations caused by foreign body involve MD.^55^ This finding leads us to favor a conservative approach in this context. However, additional studies are essential to determine the potential link between MD and the risk of foreign body perforation.

Despite the theories that agree or disagree with the management of incidentally detected MD, no conclusive evidence is available in the literature. Given the technical advancements in laparoscopic surgery, larger series and well‐designed studies are necessary to provide a more robust understanding of the optimal management strategy for incidentally detected MD. Based on review of literature, we recommend a case‐specific approach based on risk factors that increase the probability of developing symptoms throughout life. Additionally, considering the potential for MD‐related malignancy, surgical removal of the asymptomatic MD may have benefits that outweigh the risks.

In conclusion, maintaining a broad differential diagnosis is essential when dealing with abdominal pain in pediatric patients, especially in cases mimicking acute appendicitis. The perforation of MD by a foreign body, such as a wood splinter, is rare. Therefore, conducting a thorough intra‐operative assessment is crucial in patients undergoing surgery for suspected appendicitis, particularly when the appendix appears normal. Additionally, the discovery of ectopic colonic tissue in MD should be considered an area of interest to understand potential complications. A case‐specific approach is recommended to decide whether surgical resection or not of asymptomatic MD is required.

## AUTHOR CONTRIBUTIONS

**Sara Elizabeth Milla Salguero:** Conceptualization; data curation; writing – original draft; writing – review and editing. **Enrique Adalberto Medina:** Conceptualization; data curation. **Alejandra Hause Murillo:** Conceptualization; data curation. **Eduardo Smelin Perdomo Domínguez:** Conceptualization; supervision; writing – original draft; writing – review and editing.

## FUNDING INFORMATION

None.

## CONFLICT OF INTEREST STATEMENT

The authors declare that they have no conflict of interest.

## ETHICS STATEMENT

Informed consent was obtained from the patient's legal representative and all procedures were performed according to the Declaration of Helsinki.

## CONSENT

Written informed consent was obtained from the patient to publish this report in accordance with the journal's patient consent policy.

## Data Availability

None.
